# Association between exercise and risk of cardiovascular diseases in patients with non-cystic fibrosis bronchiectasis

**DOI:** 10.1186/s12931-022-02202-7

**Published:** 2022-10-18

**Authors:** Hayoung Choi, Sang Hyuk Kim, Kyungdo Han, Tai Sun Park, Dong Won Park, Ji-Yong Moon, Sang-Heon Kim, Tae-Hyung Kim, Jang Won Sohn, Ho Joo Yoon, Hyun Lee

**Affiliations:** 1 Division of Pulmonary, Allergy, and Critical Care Medicine, Department of Internal Medicine, Hallym University Kangnam Sacred Heart Hospital, Hallym University College of Medicine, Seoul, Korea; 2 Division of Molecular and Clinical Medicine, University of Dundee, Ninewells Hospital and Medical School, Dundee, UK; 3 Department of Statistics and Actuarial Science, Soongsil University, Seoul, Korea; 4 Division of Pulmonary Medicine and Allergy, Department of Internal Medicine, Hanyang University College of Medicine, Seoul, Korea; 5 Divsion of Pulmonary Medicine and Allergy, Department of Internal Medicine, Hanyang University College of Medicine, 222 Wangsimni-ro, Seongdong-gu, 04763 Seoul, Korea

**Keywords:** Bronchiectasis, Exercise, Cardiovascular diseases, Disease prevention, sedentary behavior

## Abstract

**Background:**

Although cardiovascular comorbidities negatively impact survival in patients with bronchiectasis, there is limited evidence to recommend exercise in this population. We aimed to evaluate whether exercise habit changes are related to reduced cardiovascular disease risk and explore an optimal exercise amount.

**Methods:**

This study identified 165,842 patients with newly diagnosed bronchiectasis during 2010–2016 who underwent two health examinations and were followed up until December 2020. The exposure was the change in weekly habits of moderate- or vigorous-intensity physical activity between the two examinations, classified into non-exercisers and exercisers (further classified into new exercisers, exercise dropouts, and exercise maintainers). The amount of exercise was measured using metabolic equivalents of task (MET). The outcome was the incidence of myocardial infarction (MI) or stroke.

**Results:**

During a mean of 6.2 ± 2.1 follow-up years, 4,233 (2.6%) and 3,745 (2.3%) of patients with bronchiectasis had MI or stroke, respectively. Compared to non-exercisers, exercisers had a significantly lower risk of MI or stroke by 9–28% (p < 0.001 for both). Among exercisers, exercise maintainers showed the lowest risk of MI (adjusted hazard ratio [aHR], 0.72; 95% confidence interval [CI], 0.64–0.81) and stroke (aHR, 0.72; 95% CI, 0.64–0.82) compared to non-exercisers. Regarding exercise amount, a significant risk reduction was observed only in patients with bronchiectasis who exercised for ≥ 500 MET-min/wk.

**Conclusion:**

Exercise was associated with a reduced risk of cardiovascular diseases in patients with bronchiectasis. In particular, the risk was lowest in exercise maintainers, and cardiovascular risk reduction was significant when exercising more than 500 MET-min/wk.

**Supplementary Information:**

The online version contains supplementary material available at 10.1186/s12931-022-02202-7.

## Introduction

The prevalence and disease burden of non-cystic fibrosis bronchiectasis (hereafter referred to as bronchiectasis) have been substantially increasing worldwide [1–3]. The prevalence is as high as 464–566 cases per 100,000 population [1, 2, 4, 5]. Furthermore, patients with bronchiectasis have a higher mortality risk than the general population (comparative mortality figures of 2.26 in women and 2.14 in men) [1] or than the age-, sex-, and comorbidity-matched patients without bronchiectasis (hazard ratio, 1.15) [6]. According to the literature, comorbid cardiovascular diseases largely explain higher mortality in patients with bronchiectasis [7, 8]. Hence, alleviating the risk of cardiovascular diseases can significantly reduce the long-term mortality of patients with bronchiectasis.

Despite the importance of preventing cardiovascular diseases, there is a limited evidence to recommend appropriate prevention strategies for cardiovascular comorbidities in patients with bronchiectasis. Regular exercise and physical activity are known to be effective interventions for preventing cardiovascular diseases in various populations [9]. However, there have been no studies evaluating its protective effects against cardiovascular diseases in patients with bronchiectasis. Although the current international bronchiectasis guidelines recommend regular exercise and participation in a pulmonary rehabilitation program [10, 11], the main reasons for these recommendations are to improve exercise capacity and respiratory symptoms in patients with bronchiectasis [12, 13] and not to prevent their cardiovascular comorbidities. Additionally, there is limited evidence regarding the effects of exercise and physical activity on the long-term outcomes of bronchiectasis, particularly cardiovascular comorbidities, and the optimal amount of physical activity beneficial for cardiovascular outcomes in patients with bronchiectasis. From this view, evidence-based recommendations to guide optimal exercise methods would be invaluable in preventing cardiovascular diseases and possibly improving survival in patients with bronchiectasis.

This large population-based study aimed to investigate the effects of changing the amount of physical activity on cardiovascular outcomes in patients with bronchiectasis.

## Methods

### Data source and study population

We used the National Health Insurance Service (NHIS) database of Korea, a nationwide population-based cohort [14]. The NHIS is a single-payer universal health system covering approximately 97% of the entire Korean population. In addition, the NHIS provides biennial health screening exams and examination reports on sociodemographic data, a self-questionnaire survey, clinical laboratory findings, inpatient and outpatient usage, prescription records, and recorded diagnosis based on the International Classification of Diseases 10th Revision (ICD-10) codes. More detailed information about the NHIS cohort was provided in previous studies [15, 16].

**Fig. 1:**
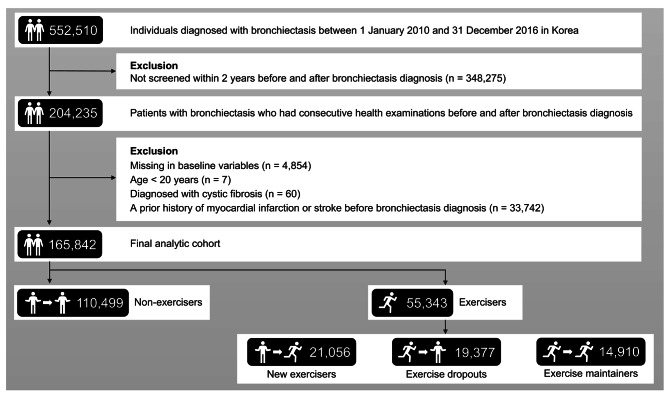
Flow chart of the study population

Among the 552,510 patients who were newly diagnosed with bronchiectasis (ICD-10 code J47) between 1 January 2010 and 31 December 2016, we included 204,235 who underwent two consecutive health examinations within two years before and after bronchiectasis diagnosis. We included those who underwent two consecutive health examinations to measure the changes in exercise habits between the two time points. Among the remaining 204,235, we excluded 4,584 with missing data, seven younger than 20 years, and 60 diagnosed with cystic fibrosis (ICD-10 code E84). Furthermore, 33,742 patients with a prior history of myocardial infarction (MI) or stroke before bronchiectasis diagnosis were excluded because this study focused on the *de novo* incidence of MI or stroke after bronchiectasis diagnosis. Finally, 165,842 patients with bronchiectasis were included in the final analytic cohort (Fig. 1).

### Exposure: change in exercise habit

To evaluate exercise habits, the intensity and frequency of physical activity were assessed using a self-report questionnaire, the International Physical Activity Questionnaire [17], during two health screening examinations performed before and after bronchiectasis diagnosis. This questionnaire has been used in several high-quality studies and contained the frequencies of weekly physical activity of varying intensities of light, moderate, or vigorous [18, 19]. Light-intensity physical activity was defined as to perform activities, such as walking slowly or vacuuming for more than 30 min. Moderate-intensity physical activity was defined as performing activities causing mild shortness of breath, such as brisk-pace walking, tennis doubles, or bicycling leisurely for more than 30 min. Vigorous-intensity physical activity was defined as performing activities causing greater shortness of breath than moderate-intensity physical activities, such as running, climbing, fast cycling, or aerobics for more than 20 min [20].

Exercise was defined as moderate- or vigorous-intensity physical activity at least once a week. To evaluate the effect of changes in exercise habits on the risk of cardiovascular diseases, the study population was subdivided into four groups according to changes in exercise performance: non-exercisers (never performed exercise in both examinations), new exercisers (non-exercisers in the first examination but exercisers in the second examination), exercise dropouts (exercisers in the first examination changed to non-exercisers in the second examination), and exercise maintainers (exercisers in both examinations). To further evaluate the overall effect of exercising habits on the risk of cardiovascular diseases compared to non-exercising habits, we grouped new exercisers, exercise dropouts, and exercise maintainers into exercisers and compared the risk of cardiovascular diseases between non-exercisers and exercisers.

The amount of exercise was also measured in this study. The energy expenditure, a minimum amount of energy consumption, was used to define the amount of exercise using metabolic equivalent of task (MET). The total MET-hours per week were calculated as the sum of the conventionally accepted intensity levels, (i.e., 2.9 METs for light-intensity exercise, 4.0 METs for moderate-intensity exercise, and 7.0 METs for vigorous-intensity exercise) [21]. For analysis, the amount of exercise was classified into < 500 MET-min/wk, 500–999 MET-min/wk, and ≥ 1000 MET-min/wk [21].

### Outcomes: incidence of cardiovascular diseases

The primary outcome was to compare the risk of incidence of cardiovascular diseases between non-exercisers and exercisers, including new exercisers, exercise dropouts, and exercise maintainers in patients with bronchiectasis. The secondary outcome was to evaluate the relationship between amount of exercise and risk of cardiovascular diseases.

As cardiovascular diseases, MI and stroke were defined using ICD-10 codes I21–I22 and I63–I64, respectively. The study population was followed until the date of the first occurrence of a cardiovascular event or the last follow-up date (31 December 2020), whichever came first (Additional file 1: Fig. S1).

### Covariates

Data for age, sex, and income were collected from the Korean NHIS database. Income level was dichotomized at the lowest 20%; the medical aid program supports individuals with a low income. Smoking status and alcohol consumption were determined by self-questionnaire. Heavy alcohol consumption was defined as more than 30 g per day. Body mass index (BMI) was calculated by dividing the weight by the square of height [22]. Blood pressure was measured using a standard mercury sphygmomanometer. Waist circumference was measured at the narrowest point between the lower rib and the iliac crest (measured to the nearest 0.1 cm). Central obesity was defined as waist circumference ≥ 90 cm in men and ≥ 85 cm in women [23]. Blood samples were collected after fasting for at least eight hours, and serum levels of glucose, total cholesterol, and creatinine were measured. The estimated glomerular filtration rate (eGFR) was calculated using the chronic kidney disease (CKD)-epidemiology collaboration equation [24].

Comorbidities that can affect the incidence of cardiovascular diseases were defined using the following ICD-10 codes: hypertension (I10–I13, I15), dyslipidemia (E78), diabetes mellitus (E10–E14), CKD (N18.1–N18.5 and N18.9), asthma (J45–J46), chronic obstructive pulmonary disease (COPD) (J42–J44, except J43.0 [unilateral emphysema]), and cancer (C00–C99 and V193) [2, 6, 25, 26].

### Statistical analysis

Data are expressed as mean ± standard deviation (SD) for continuous variables and as number (percentage) for categorical variables. Among the four groups, differences of baseline characteristics were confirmed using analysis of variance (ANOVA) for continuous variables and χ^2^ test for categorical variables. The incidence rate of cardiovascular diseases was calculated by dividing the number of incident cases by the total follow-up duration (1,000 person-years). Cox proportional hazards regression analyses were used to evaluate the associations of change and amount of exercise with incidence of MI and stroke. The multivariable model was fully adjusted for age, sex, low income, smoking status, alcohol consumption, BMI, hypertension, diabetes mellitus, dyslipidemia, asthma or COPD, and cancer. The proportional hazards assumptions were tested using graphical methods. Kaplan-Meier curves of incidence probability for incident MI or stroke were plotted according to changes in exercise habits. A two-sided p value < 0.05 was considered statistically significant. All analyses were conducted using SAS 9.3 (SAS Institute, Cary, NC, USA). 

## Results

### Baseline characteristics

In a total of 165,842 patients with bronchiectasis, the mean age was 59.8 years, and 48.4% were men. The study population comprised 66.6% non-exercisers and 33.4% exercisers. Moreover, exercisers were composed of new exercisers (n = 21,056, 12.7%), exercise dropouts (n = 19,377, 11.7%), and exercise maintainers (n = 14,910, 9.0%) according to changes in exercise habits (Fig. 1).

Exercisers were more likely to be older, male, non-smokers, and from low-income groups than non-exercisers (all p < 0.001). Regarding measurements in health screening exams, exercisers had lower total cholesterol levels, eGFR, and proportion of central obesity than non-exercisers (all p < 0.001); however, exercisers showed higher BMI, fasting blood sugar, systolic blood pressure, and longer waist circumference than non-exercisers (all p < 0.001). Of comorbidities, exercisers showed higher rates of hypertension, dyslipidemia, diabetes mellitus, and asthma or COPD compared with non-exercisers, and the differences between the four categories were statistically significant. Meanwhile, non-exercisers showed the lowest energy expenditure (32.5% for ≥ 500 MET-min/wk), followed by exercise dropouts (46.5%), new exercisers (97.8%), and exercise maintainers (98.1%) (p < 0.001) (Table 1).

**Table 1:** Baseline characteristics of the study population

Variables	Total (N = 165,842)	Non-exercisers (n = 110,499, 66.6%)	Exercisers (n = 55,343, 33.4%)			
			New exercisers (n = 21,056, 12.7%)	Exercise dropouts (n = 19,377, 11.7%)	Exercise maintainers (n = 14,910, 9.0%)	*P*-value
**Sociodemographics**						
Age, years	59.8 ± 11.9	59.5 ± 12.3	60.1 ± 11.1	60.8 ± 11.3	60.4 ± 10.6	< 0.001
< 40 years	9,621 (5.8)	7,215 (6.5)	950 (4.5)	917 (4.7)	539 (3.6)	< 0.001
40–64 years	97,408 (58.7)	64,907 (58.8)	12,625 (60.0)	10,952 (56.5)	8,924 (59.9)	
≥ 65 years	58,813 (35.5)	38,377 (34.7)	7,481 (35.5)	7,508 (38.8)	5,447 (36.5)	
Sex						< 0.001
Male	80,336 (48.4)	50,714 (45.9)	10,856 (51.6)	9,935 (51.3)	8,831 (59.2)	
Female	85,506 (51.6)	59,785 (54.1)	10,200 (48.4)	9,442 (48.7)	6,079 (40.8)	
Low income^*^	31,365 (18.9)	21,210 (19.2)	3,923 (18.6)	3,674 (19.0)	2,558 (17.2)	< 0.001
Smoking status						
Current or past smoker	24,294 (14.7)	17,239 (15.6)	2,739 (13.0)	2,526 (13.0)	1,790 (12.0)	< 0.001
Alcohol consumption						
Heavy drinker	8,402 (5.1)	5,579 (5.1)	1,093 (5.2)	865 (4.5)	865 (5.8)	< 0.001
**Measurements**						
Body mass index, kg/m^2^	23.5 ± 3.3	23.5 ± 3.3	23.6 ± 3.1	23.7 ± 3.2	23.7 ± 3.0	< 0.001
Waist circumference, cm	80.9 ± 9.1	80.8 ± 9.3	80.9 ± 8.9	81.3 ± 8.9	81.2 ± 8.6	< 0.001
Central obesity	38,903 (23.5)	26,626 (24.1)	4,653 (22.1)	4,514 (23.3)	3,110 (20.9)	< 0.001
Fasting glucose, mg/dL	100.3 ± 23.0	100.0 ± 23.1	100.4 ± 22.7	101.0 ± 23.3	101.1 ± 22.8	< 0.001
Systolic BP, mmHg	123.3 ± 14.6	123.1 ± 14.8	123.4 ± 14.4	123.8 ± 14.5	123.7 ± 14.1	< 0.001
Diastolic BP, mmHg	75.7 ± 9.5	75.7 ± 9.6	75.7 ± 9.4	75.9 ± 9.4	75.8 ± 9.4	0.057
Total cholesterol, mg/dL	193.7 ± 37.8	194.2 ± 38.0	192.7 ± 37.5	193.1 ± 37.9	192.1 ± 36.5	< 0.001
eGFR, ml/min/1.73m^2^	89.1 ± 40.7	89.5 ± 41.0	89.0 ± 44.7	87.9 ± 33.6	88.1 ± 41.5	< 0.001
**Comorbidities**						
Hypertension	64,250 (38.7)	42,287 (38.3)	8,204 (39.0)	7,884 (40.7)	5,875 (39.4)	< 0.001
Dyslipidemia	50,422 (30.4)	32,984 (29.9)	6,496 (30.9)	6,326 (32.7)	4,616 (31.0)	< 0.001
Diabetes mellitus	22,762 (13.7)	14,623 (13.2)	2,985 (14.2)	2,981 (15.4)	2,173 (14.6)	< 0.001
Chronic kidney disease	10,948 (6.6)	7,333 (6.6)	1,355 (6.4)	1,355 (7.0)	905 (6.1)	0.005
Asthma or COPD	7,885 (4.8)	4,871 (4.4)	1,204 (5.7)	975 (5.0)	835 (5.6)	< 0.001
Cancer	57,243 (34.5)	38,630 (35.0)	7,120 (33.8)	6,810 (35.1)	4,683 (31.4)	< 0.001
**Amounts of exercise**						
Energy expenditure, MET-min/wk	624.1 ± 635.2	347.0 ± 330.7	1,552.3 ± 556.7	463.0 ± 356.3	1,618.9 ± 585.2	< 0.001
≥ 500 MET-min/wk	80,102 (48.3)	35,871 (32.5)	20,599 (97.8)	9,000 (46.5)	14,632 (98.1)	< 0.001

Data are presented as number (percentage) or mean ± standard deviation

^*^Low income denotes income in the lowest 20% of the entire Korean population

*BP* blood pressure, *eGFR* estimated glomerular filtration rate, *COPD* chronic obstructive pulmonary disease, *MET* metabolic equivalent of task

### Effect of exercise on the risk of MI or stroke

**Fig. 2:**
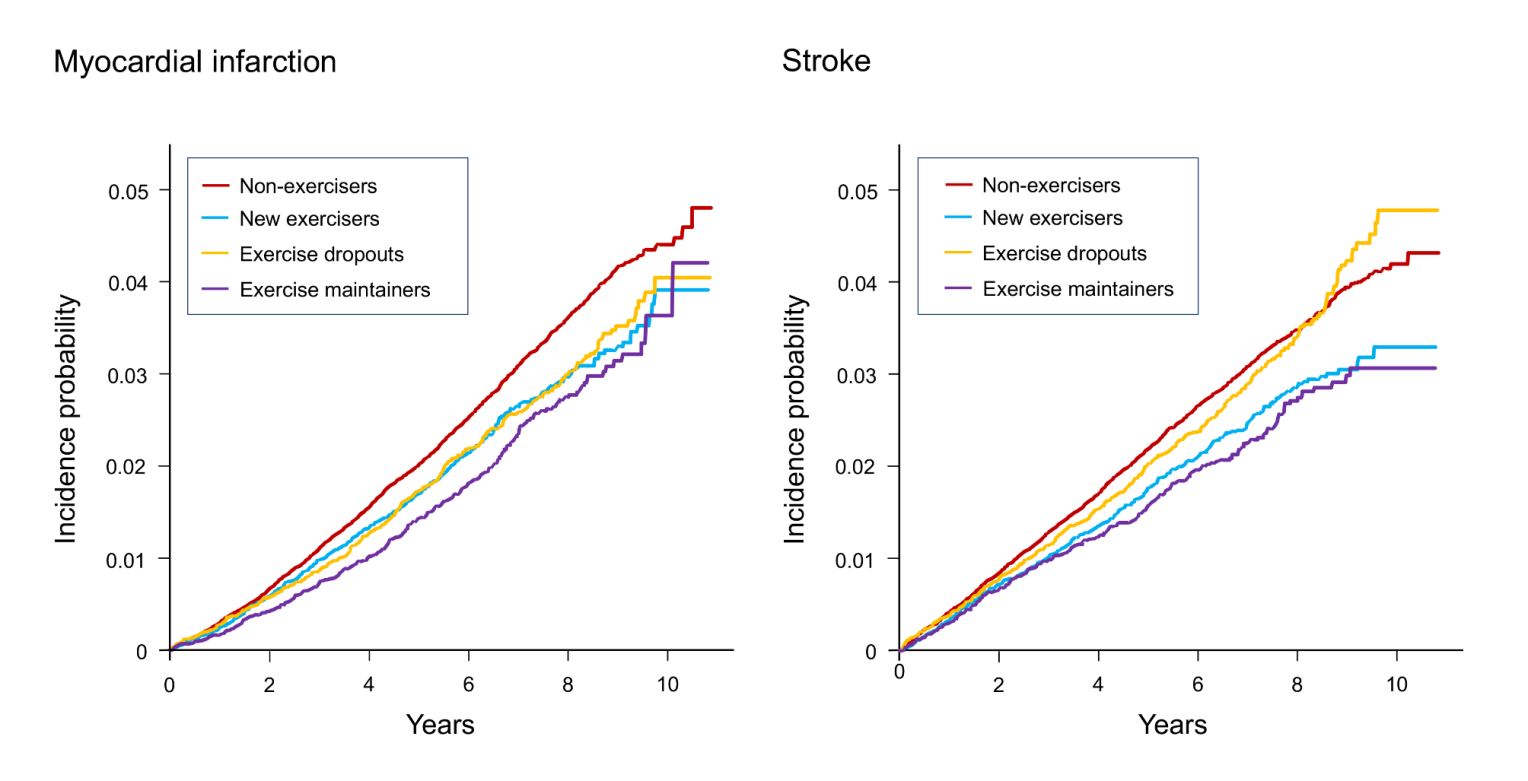
Kaplan-Meier curves of incidence probability for myocardial infarction or stroke according to change in exercise habits

During a mean follow-up duration of 6.2 ± 2.1 years, 4,233 (2.6%) and 3,745 (2.3%) patients with bronchiectasis experienced MI and stroke, respectively. The Kaplan-Meier curves showed a significantly lower incidence probability for MI in exercisers than in non-exercisers, regardless of changes in exercise habits. In contrast, the incidence probability for stroke was reduced in new exercisers and exercise maintainers, but not in exercise dropouts, compared with non-exercisers (Fig. 2).

Table 2 reveals the effect of exercise on the risk of incident MI or stroke in patients with bronchiectasis. Performing an exercise was associated with a reduced risk of 18–28% in MI or of 9–28% in stroke (p < 0.001 for both). Compared to non-exercisers, exercise maintainers showed the lowest risk of MI (adjusted hazard ratio [aHR], 0.72; 95% confidence interval [CI], 0.64–0.81), followed by exercise dropouts (aHR, 0.79; 95% CI, 0.72–0.87) and new exercisers (aHR, 0.82; 95% CI, 0.75–0.91) (p < 0.001). Regarding stroke, exercise maintainers also showed the lowest risk (aHR, 0.72; 95% CI, 0.64–0.82), followed by new exercisers (aHR, 0.79; 95% CI, 0.71–0.88) and exercise dropouts (aHR, 0.91; 95% CI, 0.82–1.00) compared with non-exercisers.

**﻿Table 2:** Effect of exercise on risk of myocardial infarction or stroke

		No. of patients	Myocardial infarction			Stroke		
			No. of events	IR (/1,000 PY)	aHR^*^ (95% CI)	No. of events	IR (/1,000 PY)	aHR^*^ (95% CI)
Overall	Non-exercisers	110,499	3,011	4.4	1 (Reference)	2,634	3.8	1 (Reference)
	Exercisers							
	New exercisers	21,056	482	3.7	0.82 (0.75–0.91)	403	3.1	0.79 (0.71–0.88)
	Exercise dropouts	19,377	444	3.7	0.79 (0.72–0.87)	447	3.7	0.91 (0.82–1.00)
	Exercise maintainers	14,910	296	3.2	0.72 (0.64–0.81)	261	2.8	0.72 (0.64–0.82)
	*P*-value				< 0.001			< 0.001
Age, years								
< 40 years	Non-exercisers	7,215	35	0.7	1 (Reference)	14	0.3	1 (Reference)
	Exercisers							
	New exercisers	950	1	0.2	0.22 (0.03–1.62)	1	0.2	0.56 (0.07–4.23)
	Exercise dropouts	917	7	1.1	1.53 (0.68–3.45)	4	0.6	2.21 (0.73–6.71)
	Exercise maintainers	539	2	0.6	0.78 (0.19–3.26)	2	0.6	1.94 (0.44–8.55)
	*P*-value				0.228			0.479
40–64 years	Non-exercisers	64,907	1,301	3.1	1 (Reference)	811	2.0	1 (Reference)
	Exercisers							
	New exercisers	12,625	229	2.8	0.87 (0.76–1.00)	147	1.8	0.88 (0.74–1.05)
	Exercise dropouts	10,952	183	2.6	0.79 (0.67–0.92)	137	2.0	0.94 (0.78–1.12)
	Exercise maintainers	8,924	138	2.4	0.76 (0.64–0.90)	68	1.2	0.58 (0.45–0.74)
	*P*-value				< 0.001			< 0.001
≥ 65 years	Non-exercisers	38,377	1,675	7.5	1 (Reference)	1,809	8.1	1 (Reference)
	Exercisers							
	New exercisers	7,481	252	5.7	0.79 (0.69–0.90)	255	5.8	0.75 (0.65–0.85)
	Exercise dropouts	7,508	254	5.8	0.78 (0.69–0.90)	306	7.0	0.88 (0.78–1.00)
	Exercise maintainers	5,447	156	4.9	0.69 (0.59–0.82)	191	6.0	0.79 (0.68–0.91)
	*P*-value				< 0.001			< 0.001
	*P* for interaction^†^				0.434			0.074
Sex								
Male	Non-exercisers	50,714	1,550	4.9	1 (Reference)	1,430	4.6	1 (Reference)
	Exercisers							
	New exercisers	10,856	295	4.4	0.84 (0.74–0.95)	255	3.8	0.78 (0.68–0.89)
	Exercise dropouts	9,935	262	4.3	0.79 (0.69–0.90)	280	4.6	0.89 (0.79–1.02)
	Exercise maintainers	8,831	203	3.7	0.73 (0.63–0.84)	197	3.6	0.75 (0.65–0.87)
	*P*-value				< 0.001			< 0.001
Female	Non-exercisers	59,785	1,461	3.9	1 (Reference)	1,204	3.2	1 (Reference)
	Exercisers							
	New exercisers	10,200	187	2.9	0.79 (0.68–0.93)	148	2.3	0.82 (0.69–0.97)
	Exercise dropouts	9,442	182	3.1	0.79 (0.68–0.92)	167	2.8	0.93 (0.79–1.09)
	Exercise maintainers	6,079	93	2.5	0.71 (0.58–0.88)	64	1.7	0.65 (0.51–0.84)
	*P*-value				< 0.001			0.002
	*P* for interaction^†^				0.946			0.714

^*^In the adjustment, age, sex, low income, smoking status, alcohol consumption, body mass index, hypertension, diabetes mellitus, dyslipidemia, asthma or COPD, and cancer were included

^†^The *P* for interaction was calculated by adding an interaction term to the multivariable model

*IR* incidence rate, *PY* person-years, *aHR* adjusted hazard ratio, *CI* confidence interval, *COPD* chronic obstructive pulmonary disease

In stratified analysis, age or sex did not have a significant interaction with the association between exercise and cardiovascular outcomes. The risk-reducing effect of exercise for MI or stroke was only significant in those ≥ 40 years of age (p < 0.001 for both 40–64 years and ≥ 65 years). The effect of exercise was not significantly different between men and women, and exercisers of both genders showed a significant reduction in the risk of MI (p < 0.001 for both) or stroke (p < 0.001 for men; p = 0.002 for women) (Table 2).

### Amount of exercise and risk of MI or stroke

The associations between amount of exercise, measured by energy expenditure using METs, and risk of MI or stroke in patients with bronchiectasis are described in Table 3. Exercisers showed a lower risk for MI or stroke than non-exercisers regardless of the amount of exercise (p < 0.001 for both). However, a significant reduction was observed only in those with ≥ 500 MET-min/wk.

**﻿Table 3:** The relationship between amount of exercise and risk of myocardial infarction or stroke

	No. of patients	Myocardial infarction			Stroke		
		No. of events	IR (/1,000 PY)	aHR^*^ (95% CI)	No. of events	IR (/1,000 PY)	aHR^*^ (95% CI)
Non-exercisers	110,499	3,011	4.4	1 (Reference)	2,634	3.8	1 (Reference)
Overall exercisers							
< 500 MET-min/wk	11,112	302	4.3	0.91 (0.81–1.03)	287	4.1	0.99 (0.88–1.12)
500–999 MET-min/wk	11,407	228	3.2	0.71 (0.62–0.82)	222	3.1	0.80 (0.69–0.91)
≥ 1000 MET-min/wk	32,824	692	3.4	0.76 (0.70–0.83)	602	3.0	0.76 (0.69–0.83)
*P*-value				< 0.001			< 0.001
Non-exercisers	110,499	3,011	4.4	1 (Reference)	2,634	3.8	1 (Reference)
New exercisers							
< 500 MET-min/wk	457	11	3.8	0.89 (0.49–1.61)	14	4.8	1.38 (0.82–2.33)
500–999 MET-min/wk	2,384	58	3.8	0.92 (0.71–1.19)	35	2.3	0.66 (0.48–0.93)
≥ 1000 MET-min/wk	18,215	413	3.6	0.81 (0.73–0.90)	354	3.1	0.79 (0.71–0.89)
*P*-value				< 0.001			< 0.001
Non-exercisers	110,499	3,011	4.4	1 (Reference)	2,634	3.8	1 (Reference)
Exercise dropouts							
< 500 MET-min/wk	10,377	284	4.4	0.91 (0.81–1.03)	269	4.1	0.99 (0.87–1.12)
500–999 MET-min/wk	7,520	142	3.1	0.66 (0.56–0.78)	158	3.4	0.83 (0.71–0.98)
≥ 1000 MET-min/wk	1,480	18	2.0	0.51 (0.32–0.81)	20	2.2	0.67 (0.43–1.04)
*P*-value				< 0.001			0.046
Non-exercisers	110,499	3,011	4.4	1 (Reference)	2,634	3.8	1 (Reference)
Exercise maintainers							
< 500 MET-min/wk	278	7	3.8	0.93 (0.44–1.94)	4	2.2	0.64 (0.24–1.71)
500–999 MET-min/wk	1,503	28	2.9	0.68 (0.47–0.98)	29	3.1	0.82 (0.57–1.18)
≥ 1000 MET-min/wk	13,129	261	3.2	0.72 (0.64–0.82)	228	2.8	0.72 (0.62–0.82)
*P*-value				< 0.001			< 0.001

^*^In the adjustment, age, sex, low income, smoking status, alcohol consumption, body mass index, hypertension, diabetes mellitus, dyslipidemia, asthma or COPD, and cancer were included

*IR* incidence rate, *PY* person-years, *aHR* adjusted hazard ratio, *CI* confidence interval, *MET* metabolic equivalent of task, *COPD* chronic obstructive pulmonary disease

Regarding MI, compared to non-exercisers, exercisers with 500–999 MET-min/wk showed the lowest risk (aHR, 0.71; 95% CI, 0.62–0.82), followed by exercisers with ≥ 1000 MET-min/wk (aHR, 0.76; 95% CI, 0.70–0.83) and exercisers with < 500 MET-min/wk (aHR, 0.91; 95% CI, 0.81–1.03). Subgroup analysis showed a significantly reduced risk for MI in new exercisers with ≥ 1000 MET-min/wk and in exercise dropouts and exercise maintainers with ≥ 500 MET-min/wk (all p < 0.001). The greatest risk reduction for MI was 49%, observed in exercise dropouts with initial ≥ 1000 MET-min/wk (Table 3).

For stroke, compared to non-exercisers, exercisers with ≥ 1000 MET-min/wk showed the lowest risk (aHR, 0.76; 95% CI, 0.69–0.83), followed by exercisers with 500–999 MET-min/wk (aHR, 0.80; 95% CI, 0.69–0.91) and exercisers with < 500 MET-min/wk (aHR, 0.99; 95% CI, 0.88–1.12). In subgroup analysis, a significantly reduced risk for stroke was observed in new exercisers with ≥ 500 MET-min/wk (p < 0.001), in exercise dropouts with 500–999 MET-min/wk (p = 0.046), and in exercise maintainers with ≥ 1000 MET-min/wk (p < 0.001). The greatest risk reduction for stroke was 44%, observed in new exercisers with 500–999 MET-min/wk (Table 3).

## Discussion

In this large-scale population-based cohort study, notable findings are as follows. First, more than 2% of patients with bronchiectasis developed MI or stroke during a mean of 6.2 follow-up years. Second, approximately two-thirds of patients with bronchiectasis have remained non-exercisers after their diagnosis of bronchiectasis. Third, exercise in patients with bronchiectasis was associated with a lower risk of MI or stroke. The effect of exercise on cardiovascular risk reduction was highest in exercise maintainers. This effect was also significant in those who initiated exercise after being diagnosed with bronchiectasis. Finally, exercising more than 500 MET-min/wk reduced the risk of cardiovascular diseases. Our findings suggest that a proper amount of regular exercise could prevent cardiovascular diseases in patients with bronchiectasis.

Evidence supports increased cardiovascular risk in patients with bronchiectasis [6, 7, 27]. Although the reasons for increased cardiovascular risk in patients with bronchiectasis remain unclear, endothelial dysfunction is one of the proposed mechanisms [28]. Endothelial damage can be directly induced by increased platelet activation and systemic inflammation in patients with bronchiectasis [29]. Moreover, the increased cardiovascular risk was more pronounced after a respiratory tract infection in patients with bronchiectasis compared with their baseline (1.56 of incident rate ratio in the 91 days) [7]. Consequently, we need interventions to modify cardiovascular risk in patients with bronchiectasis during their stable state. Some studies demonstrated that exercise could alter catecholamine secretion, signaling protein expression and endothelial cell metabolism, leading to endothelial repair [30, 31]. In the same vein, the current study also revealed that exercise was related to reduced cardiovascular risk in patients with bronchiectasis. Therefore, future bronchiectasis guidelines should include a recommendation for exercise to reduce the risk of cardiovascular diseases.

In this study, exercise was associated with reduced cardiovascular disease risk in patients with bronchiectasis; risk of MI and stroke was reduced by up to 28% in exercise maintainers. Additionally, the effect of exercise is significant for patients who initiated an exercise program after being diagnosed with bronchiectasis. Also, a previous study revealed that low levels of physical activity and high sedentary time were associated with a 5.9-fold higher risk of hospitalization due to bronchiectasis exacerbation [32], which is a well-known risk factor for long-term mortality [33]. Taken together, these findings suggest that exercise has positive effects on long-term survival in patients with bronchiectasis via two mechanisms: reduced cardiovascular disease risk and reduced bronchiectasis exacerbation. In the near future, clinicians might have to evaluate the exercise status of patients with bronchiectasis and prescribe exercise to improve the long-term survival of their patients.

Notably, exercisers were older, had a higher proportion of males, higher BMI, higher fasting blood glucose, and higher systolic blood pressure than non-exercisers. The aforementioned factors, such as older age, male sex, BMI, diabetes mellitus, and hypertension, are well-established determinants of cardiovascular disease risk [34–37]. Despite having many risk factors for cardiovascular diseases, patients with bronchiectasis who exercise revealed a significantly lower cardiovascular risk than those who did not exercise even before adjusting for those factors (see incidence rate of MI or stroke in Table 2), which might be an important finding for clinicians. When patients are diagnosed with bronchiectasis, respiratory physicians cannot modify some cardiovascular risk determinants, such as sex and comorbidities; however, they may be able to modify exercise habits and sedentary behavior to improve long-term prognosis in patients with bronchiectasis.

Regarding the amount of exercise, more than 500 MET-min/wk was associated with a lower risk of cardiovascular diseases in patients with bronchiectasis. These results were consistent with the World Health Organization Guidelines on Physical Activity and Sedentary Behaviour, suggesting regular exercise more than 500 MET-min/wk for adults to achieve health benefits [38]. Regardless of the change in exercise habits, the risk of MI and stroke was reduced in those who exercised more than the recommended amount (500 MET-min/wk). In line with those results, a previous study of 64 patients with bronchiectasis revealed that reduced physical activity (< 6,290 steps/day) or high sedentary behavior (≥ 7.8 h/day) were related to a higher risk of hospitalization due to bronchiectasis exacerbation [32]. However, the optimal amount of exercise in patients with bronchiectasis is not known, and future studies are warranted to reveal this issue.

To the best of our knowledge, this is the first study evaluating whether exercise reduces the risk of cardiovascular diseases in patients with bronchiectasis and exploring the optimal physical activity in the population. Another strength of this study was the inclusion of various covariates such as BMI, smoking status, alcohol consumption, and comorbidities. However, this study has several limitations that should be acknowledged. First, the intensity and frequency of physical activity were evaluated by self-reported questionnaires that could be influenced by recall bias. Second, physiologic parameters such as peak heart rate and oxygen uptake could not be included to evaluate exercise intensity. Future research using more objective parameters on exercise intensity is required. Third, assessment for bronchiectasis severity was not available. Bronchiectasis is a chronic respiratory disease that affects workout abilities, and the effect of exercise can vary depending on the severity of bronchiectasis. When prescribing exercise in real practice, the exercise capacity of the patient with bronchiectasis should be considered. Fourth, this study was conducted in a single Asian country with a limited ethnic diversity. Therefore, generalization of our findings to other ethnic groups should be done with caution. Fifth, because our dataset lacked data on energy consumption, we could not consider the effect of energy consumption, an important factor affecting cardiovascular risks [39], on the relationship between physical activity and cardiovascular disease.

## Conclusion

Exercise was associated with reduced risk of cardiovascular diseases in patients with bronchiectasis. In particular, risk was lowest in exercise maintainers, and cardiovascular risk reduction was significant in those who exercised more than 500 MET-min/wk. Our study provides evidence of exercise prescription for patients with bronchiectasis to prevent cardiovascular diseases.

## Electronic supplementary material

Below is the link to the electronic supplementary material.

Supplementary Material 1

## Data Availability

The datasets used and analyzed during the current study are available from the corresponding author on reasonable request.

## Declarations

**Ethnics approval and consent to participate**.

The study protocol was approved by the Institutional Review Board of Hallym University Kangnam Sacred Heart Hospital (application no. 2022-01-025). The requirement for informed consent was waived because the NHIS database was constructed after anonymization of patients.

### Figure S1

Study design schema. ^*^within two years before bronchiectasis diagnosis. ^†^within two years after bronchiectasis diagnosis.

*MI* myocardial infarction.

## List of abbreviations

NHIS: National Health Insurance Service.
ICD-10: International Classification of Diseases 10th Revision.
MI: myocardial infarction.
MET: metabolic equivalent of task.
BMI: body mass index.
eGFR: estimated glomerular filtration rate.
CKD: chronic kidney disease.
COPD: chronic obstructive pulmonary disease.
SD: standard deviation.
ANOVA: analysis of variance.
aHR: adjusted hazard ratio.
CI: confidence interval.
