# Supplementary figures and images for: Association between exercise and risk of cardiovascular diseases in patients with non-cystic fibrosis bronchiectasis
Source: Respir Res. 2022 Oct 18;23:288. doi: 10.1186/s12931-022-02202-7 (PMC9580142; doi:10.1186/s12931-022-02202-7)

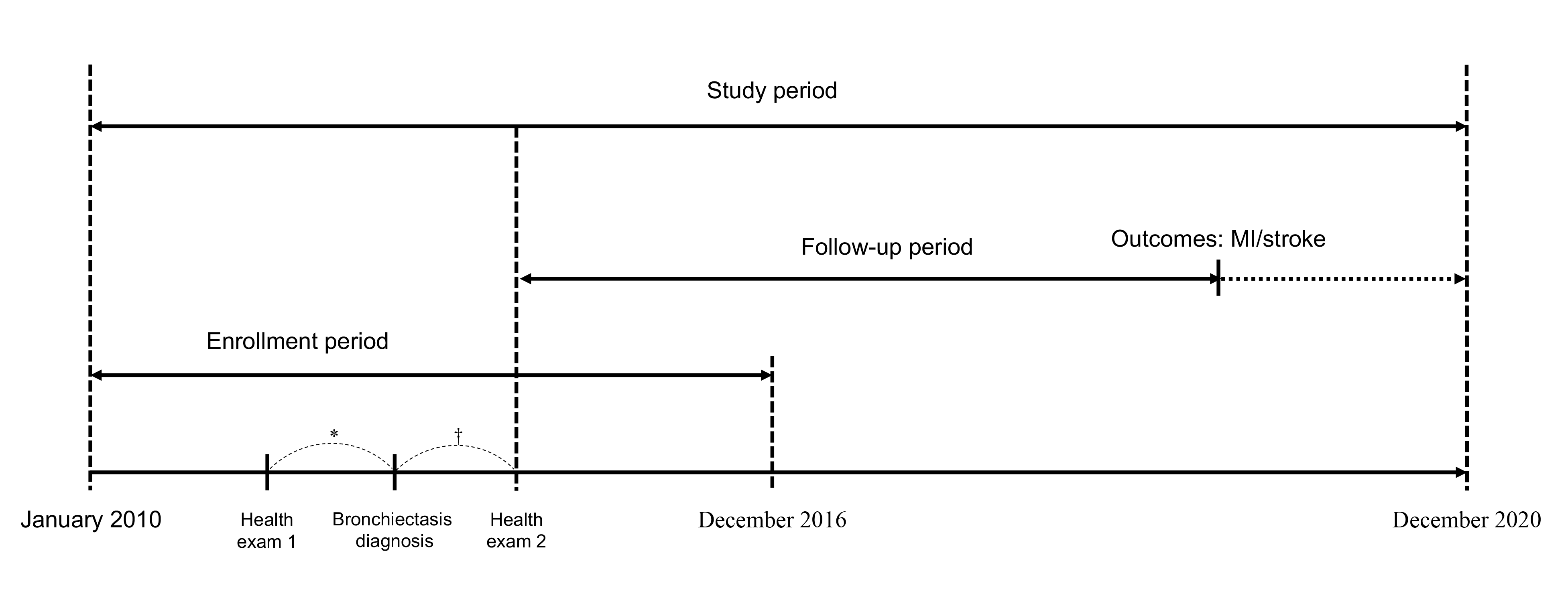

Supplement: Supplementary file 1 — Supplementary Material 1 [file 12931_2022_2202_MOESM1_ESM.png]
